# Prediction of the risk of coronary arterial lesions in Kawasaki disease by serum 25-hydroxyvitamin D_3_

**DOI:** 10.1007/s00431-014-2346-y

**Published:** 2014-06-03

**Authors:** Yan-Li Chen, Juan-Li Wang, Wei-Qin Li

**Affiliations:** 1 Department of Pediatrics, Affiliated Hospital of Xi’an Medical College, Number 48 Fenghao West Road, Xi’an, 710077 China; 2 Department of Cardiology, Children’s Hospital of Xi’an, Xi’an, 710003 China

**Keywords:** Kawasaki disease, 25-Hydroxyvitamin D_3_, Coronary artery lesions

## Abstract

Kawasaki disease (KD) is associated with the development of coronary arterial lesions (CALs) in children. We aimed to test the hypothesis that circulating 25-hydroxyvitamin D_3_ [25-(OH)D_3_] could be identified as a clinical parameter for predicting CALs secondary to KD in children. We enrolled 35 children with KD in the acute phase and measured serum 25-(OH)D_3_ levels in all of them, then followed up by echocardiography for CALs. Additionally, serum 25-(OH)D_3_ levels were obtained in 23 febrile children with respiratory tract infections and 30 healthy children. Of the 35 KD children, nine had CALs according to echocardiography and 26 did not (NCALs). Serum 25-(OH)D_3_ levels were not significantly different between NCALs and healthy children (49.2 ± 23.8 versus 44.1 ± 30.2 ng/ml; *P* = 0.49). Serum 25-(OH)D_3_ levels were significantly higher in children with CALs than those without CALs (83.9 ± 26.3 versus 49.2 ± 23.8 ng/ml; *P* = 0.001). The cutoff value of 65 ng/ml to predict subsequent CALs had a specificity of 0.73, sensitivity of 0.78, and diagnostic accuracy of 0.74. *Conclusion*: Serum 25-(OH)D_3_ levels were elevated dur-ing the acute phase in KD children who had subsequent CALs. Serum 25-(OH)D_3_ levels in the acute phase of KD may be used to predict subsequent CALs.

## Introduction

Kawasaki disease (KD), first described in 1967 by Dr. Tomisaku Kawasaki as mucocutaneous lymph node syndrome [7], is an acute systemic vasculitis that primarily affects small- and medium-sized arteries. KD that predominantly affects children younger than 5 years is mostly a self-limiting disease, but sometimes gives rise to serious cardiac complications. Coronary arterial lesions (CALs), including dilatations and aneurysms, are identified as the most serious complications of KD. In China, approximately 8.5 to 20.6 % of KD patients develop CALs [4, 11, 12, 16, 28]. Even after high-dose immunoglobulin therapy, 5–10 % of KD children develop CALs [14]. CALs secondary to KD are associated with changes in certain biomarkers, such as platelet count, neutrophil count, plateletcrit, platelet distribution width, mean platelet volume, erythrocyte sedimentation rate, cardiac troponin I, endothelin-1, albumin, and hemoglobin [3] . In a retrospective study [2] involving 113 Chinese children, Kobayashi scoring system was applied to predict coronary arterial lesions in KD, and the results showed that the sensitivity of the Kobayashi scoring system was 0.56. Though the Kobayashi scoring system had the ability to predict the coronary arterial lesions in KD patients, the power was not high enough. Therefore, a laboratory marker in the initial acute stage of KD that could predict the development of subsequent CALs is very much desired.

The exact mechanisms underlying the development of CALs secondary to KD remain poorly understood. Several markers of immune activation are increased in KD patients, suggesting their involvement in the pathogenesis [8, 19, 27]. Hyperactive immune cells, especially T cells, with excessive cytokines may be responsible for tissue injury in KD [10]. Either dietary vitamin D or that produced in the skin is transported to the liver for conversion to 25-hydroxyvitamin D_3_ [25-(OH)D_3_]. Circulating 25-(OH)D_3_ is the precursor of the active 1,25-dihydroxyvitamin D_3_ (1,25-(OH)_2_D_3_). Almost every tissue in the human body carries receptors for 1,25-(OH)_2_D_3_ (vitamin D receptor, VDR), which affects epithelial cell, T cell, B cell, and dendritic cell functions. In addition, VDR has been identified in the immune system [1, 5, 23, 25]. Adjunctive 1-alpha, 25-dihydroxyvitamin D_3_ therapy exhibiting anti-inflammatory and immunomodulatory effects during KD vasculitis have been reported [9, 20]. The expression of VDR in T cells was higher in KD patients than febrile children with respiratory tract infections and healthy children [17]. Over activated T cells might pro-mote 25-(OH)D_3_ release. Therefore, more intense inflammatory response in CALs’ patients may have increased VDR expression and subsequently elevated 25-(OH)D_3_ levels. In addition, vitamin D deficiency as an indepen-dent risk factor for arterial disease has been reported [22].

Taken together, we hypothesized that CALs induce excessive vascular calcification; calcifications would stimulate CYP27B1 enzyme expression and in turn increase serum 25-(OH)D_3_ levels. The purpose of this study was to investigate the clinical value of serum 25-(OH) D_3_ levels as a predictor of CALs in children with KD.

## Material and methods

### Study population

We enrolled 35 children diagnosed with KD [15] between March 2011 and July 2013 at the Affiliated Hospital of Xi’an Medical College. All the children received intravenous immune globulin treatment. Once Kawasaki disease is diagnosed, immunoglobulin therapy should begin within 24 h. Echocardiography was used to detect the presence of CALs during the course of the disease. Of the 35 children diagnosed with KD, nine (six boys and three girls) developed CALs based on echocardiographic results and 26 (19 boys and 7 girls) did not develop CALs (NCALs). In addition, 23 children with respiratory tract infections were included as febrile controls. These children did not have cutaneous eruption or myocardial injury and were excluded if they had abnormal myocardial enzyme levels or an abnormal electrocardiogram. Furthermore, 30 healthy children were selected as normal controls. Children with heart, lung, or kidney disease were excluded. The study was approved by the Ethics Committee of the Affiliated Hospital of Xi’an Medical College. Parental informed consent was obtained for each child enrolled in this study. There were no significant differences between the groups regarding baseline characteristics.

### Measurement of serum 25-(OH)D_3_ levels

Fasting blood samples (approximately 3 ml) were collected for each patient between 5 and 10 days of disease onset. The samples were centrifuged at 3,000 rpm/min for 10 min and stored at −70 °C until analysis. Serum 25-(OH)D_3_ concentrations were measured using a commercially available ELISA kit (Shanghai XinLe Biological Technology Co., Ltd.) according to the manufacturer’s instructions. The same method was used to measure 25-(OH) D_3_ levels in febrile children and healthy control children.

### Statistical analysis

Continuous data were expressed as mean±standard deviation (SD) if the values were normally distributed. Statistical comparisons of continuous variables were performed by a two-tailed Student’s *t* test. A χ^2^ test was used to compare categorical variables. *P* < 0.05 was considered statistically significant. All analyses were performed with SPSS V.17.0 (SPSS Inc, Chicago, Illinois, USA). To compare the power of serum level of 25-(OH)D_3_ levels in predicting CALs, receiver-operating characteristic curves (ROCs) were plotted and areas under the curve (AUCs) were calculated

## Results

The KD cases included 26 boys and 9 girls with an age range of 4 months to 4 years. Six boys and three girls developed CALs. The febrile children group included 11 boys and 12 girls with an age range of 5 months to 4 years. The normal controls consisted of 16 boys and 14 girls with an age range of 5 months to 4 years.

### Comparison of 25-(OH)D_3_ levels

The mean serum 25-(OH)D_3_ levels in febrile children was significantly lower than healthy children (27.9 ± 20.6 versus 44.1 ± 30.2 ng/ml; *P* = 0.025). This level was not significantly different between NCALs and healthy children (49.2 ± 23.8 versus 44.1 ± 30.2 ng/ml; *P* = 0.49) (Table 1). In addition, the mean serum 25-(OH)D_3_ levels were significantly higher in children with CALs than those without CALs (83.9 ± 26.3 versus 49.2 ± 23.8 ng/ml; *P* = 0.001).

**Table 1:** Comparison of serum 25-(OH)D_3_ levels between different groups

Group	Number	25-(OH)D_3_ (ng/ml)
Febrile children with respiratory tract infections	23	27.9 ± 20.2
Healthy control	30	44.1 ± 30.2^a^
NCALs	26	49.2 ± 23.8^b^
CALs	9	83.9 ± 26.3^c^

Values were expressed as mean±SD

*CALs* coronary arterial lesions, *NCALs* no coronary arterial lesions

^a^Comparing with febrile children with respiratory tract infection group: *t* = 2.31 *P* = 0.025

^b^Comparing with healthy control group: *t* = 1.16, *P* = 0.49

^c^Comparing with NCALs group: *t* = 3.68, *P* = 0.001

### ROCs

**Fig. 1:**
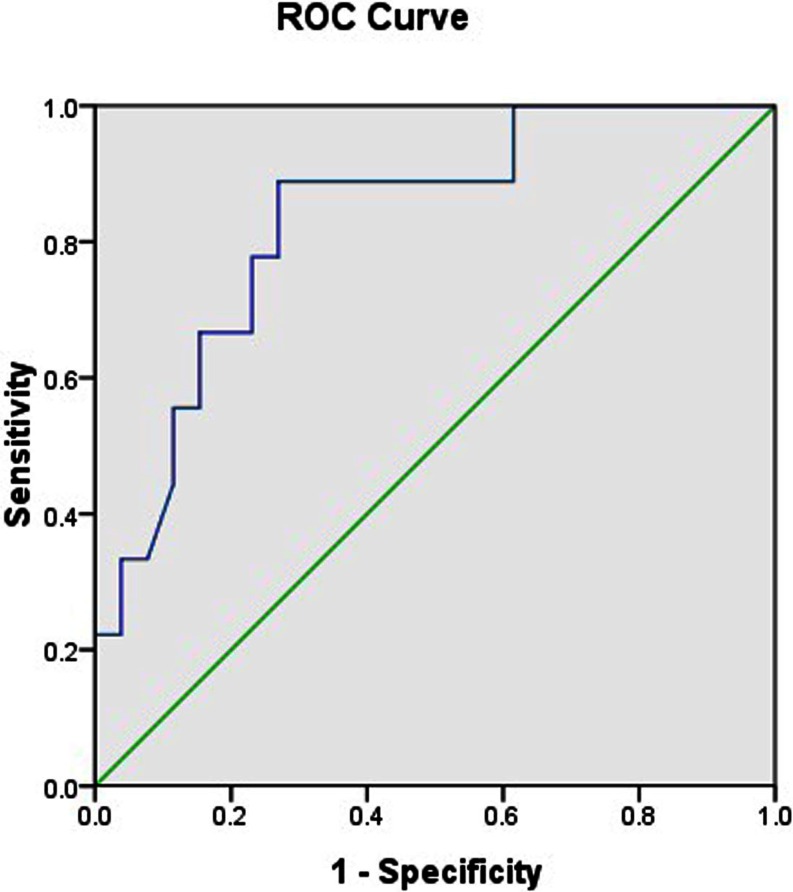
Predictive performance of the serum 25-(OH)D_3_. ROCs of serum 25-(OH)D_3_ levels used to predict coronary arterial lesions in children diagnosed with Kawasaki disease. The area under the curve of serum 25-(OH)D_3_ was 0.831

To assess the performance of serum level of 25-(OH)D_3_ in predicting CALs, ROCs were plotted and the AUCs was calculated. As shown in Fig. 1, the AUC for predicting CALs of 25-(OH)D_3_ was 0.831. When the cutoff value of 65 ng/ml for 25-(OH)D_3_ based on the ROC was obtained for predicting CALs, it generated a sensitivity of 78 % and specificity of 73 %. Detailed characteristics of cutoff value of 65 ng/ml for CAL prediction were listed in Table 2.

**Table 2:** Performance of 25-(OH)D_3_ with a cutoff level of 65 ng/ml

Parameters	Value (%)
Sensitivity	78
Specificity	73
Positive predictive value	50
Negative predictive value	90
Diagnostic accuracy	74

## Discussion

The current study showed that serum levels of 25(OH)D_3_ were significantly higher in KD children who developed CALs compared with children who did not develop CALs. Higher serum 25-(OH)D_3_ levels in the acute phase of KD was associated with an increased the risk of developing CALs. A cutoff value of 65 ng/ml had a specificity of 0.73 and sensitivity of 0.78 for predicting CALs. These results indicate that 25-(OH)D_3_ has a potential role in the pathogenesis of CALs in children with KD.

Early identification of the risk of CALs in KD is very important to formulate a therapeutic strategy. A well-designed meta-analysis [3] summarized 16 biomarkers associated with CALs in Chinese KD children. The results indicated that the biomarkers, including platelet count, neutrophil count, plateletcrit, platelet distribution width, mean platelet volume, erythrocyte sedimentation rate, cardiac troponin I, endothelin-1, albumin, and hemoglobin, were associated with developing CALs among Chinese KD children. However, no generally accepted specific biomarkers for predicting CALs in clinical practice have been established. In addition, serum NT pro-B-type natriuretic peptide above 1,000 pg/ml [6] or serum sodium less than 135 mEq/l [13] has been used to diagnose CALs secondary to KD. More recently, serum interleukin-18 was also used to identify CALs in children with KD[24].

To the best of our knowledge, the association of serum 25-(OH)D_3_ levels in the diagnosis of CALs among children with KD has not been reported, and this is the first report to identify the role of 25(OH)D_3_ in predicting CALs in KD. We found that serum 25-(OH)D_3_ levels measured in the acute phase KD was significantly higher in children with CALs than those without CALs (*P* = 0.001). A 25-(OH)D_3_ cutoff point of 65 ng/ml had a sensitivity of 0.78 and specificity of 0.73 for predicting the development of subsequent CALs. Therefore, measurement of 25-(OH)D_3_ levels in the acute phase KD could be beneficial in clinical practice to identify cardiac complications. However, 25-(OH)D_3_ levels were not significantly different between the NCAL group and healthy children, making it difficult to be used as a parameter to exclude NCALs in KD. The mechanisms involved in the elevation of serum 25-(OH)D_3_ levels in the acute phase of KD need to be explored further. The proposed mechanism include the higher expression of VDR in T cells in KD patients than febrile children with respiratory tract infections and healthy children. Overactivated T cells likely promote 25-(OH)D_3_ release. Therefore, more intense inflammatory response in CALs’ patients may have increased VDR expression and subsequently elevated 25-(OH)D_3_ levels.

The newborn in China receive vitamin D supplement after day 14 of birth regularly without measurement of serum 25-(OH)D level. The dosage for under 1 year old is 500 U per day and for 1 to 2 years old is 600 U per day until 18 years old. However, due to the vitamin D preparation available in the market, almost all the children received vitamin D supplement under 2 years old and few children more than 2 years old received supplement vitamin D [26] .

There were some limitations in this study. The small sample size of KD children lowered the statistical power. The high incidence of the occurrence of CALs in this study might be correlated with the small sample size. Second, the prevalence of CALs in the KD children directly influenced the predictive values. Further, 25-(OH)D_3_ levels may vary according to race [18] and region [21], so caution is needed before the current results are generalized to other patients. Third, the severity of KD is classified by the severity of coronary artery dilatation. However, due to the small sample size, we did not conduct correlation analysis between the severity of CALs and 25-(OH)D_3_ level; therefore, the relationships are still unclear between the severity of CALs and 25-(OH)D_3_ level. Finally, since 25-(OH)D_3_ levels were not elevated in KD children with normal coronary arteries in the current study, serum 25-(OH)D_3_ levels may not be used to diagnose KD.

## Conclusions

Serum 25-(OH)D_3_ levels greater than 65 ng/ml in the acute phase of KD may be predictive of developing subsequent CALs; however, it may not be helpful in the diagnosis of KD. More studies are needed to confirm whether serum 25-(OH)D_3_ levels could be used to predict CALs in KD.

## Abbreviations

CALs: Coronary arterial lesions
KD: Kawasaki disease

## References

[1] Cantorna MT, Zhu Y, Froicu M, Wittke A (2004). Vitamin D status, 1,25-dihydroxyvitamin D3, and the immune system. Am J Clin Nutr.

[2] Chen JJ, Liu YL (2011). Clinical values of Kobayashi scoring system in Chinese children with Kawasaki disease. Acta Med Univ Sci Technol Huazhong.

[3] Chen J, Liu Y, Liu W, Wu Z (2011). A meta-analysis of the biomarkers associated with coronary artery lesions secondary to Kawasaki disease in Chinese children. J Huazhong Univ Sci Technol Med Sci.

[4] Du ZD, Zhao D, Du J, Zhang YL, Lin Y, Liu C, Zhang T (2007). Epidemiologic study on Kawasaki disease in Beijing from 2000 through 2004. Pediatr Infect Dis J.

[5] Hewison M (2010). Vitamin D and the intracrinology of innate immunity. Mol Cell Endocrinol.

[6] Kaneko K, Yoshimura K, Ohashi A, Kimata T, Shimo T, Tsuji S (2011). Prediction of the risk of coronary arterial lesions in Kawasaki disease by brain natriuretic peptide. Pediatr Cardiol.

[7] Kawasaki T (1967). Acute febrile mucocutaneous syndrome with lymphoid involvement with specific desquamation of the fingers and toes in children. Arerugi.

[8] Kemmotsu Y, Saji T, Kusunoki N, Tanaka N, Nishimura C, Ishiguro A, Kawai S (2012). Serum adipokine profiles in Kawasaki disease. Mod Rheumatol.

[9] Kudo K, Hasegawa S, Suzuki Y, Hirano R, Wakiguchi H, Kittaka S, Ichiyama T (2012). 1alpha,25-Dihydroxyvitamin D(3) inhibits vascular cellular adhesion molecule-1 expression and interleukin-8 production in human coronary arterial endothelial cells. J Steroid Biochem Mol Biol.

[10] Lee KY, Rhim JW, Kang JH (2012). Kawasaki disease: laboratory findings and an immunopathogenesis on the premise of a "protein homeostasis system". Yonsei Med J.

[11] Li XH, Li XJ, Li H, Xu M, Zhou M (2008). Epidemiological survey of Kawasaki disease in Sichuan province of China. J Trop Pediatr.

[12] Ma XJ, Yu CY, Huang M, Chen SB, Huang MR, Huang GY (2010). Epidemiologic features of Kawasaki disease in Shanghai from 2003 through 2007. Chin Med J (Engl).

[13] Nakamura Y, Yashiro M, Uehara R, Watanabe M, Tajimi M, Oki I, Ojima T, Sonobe T, Yanagawa H (2004). Use of laboratory data to identify risk factors of giant coronary aneurysms due to Kawasaki disease. Pediatr Int.

[14] Newburger JW, Takahashi M, Beiser AS, Burns JC, Bastian J, Chung KJ, Colan SD, Duffy CE, Fulton DR, Glode MP (1991). A single intravenous infusion of gamma globulin as compared with four infusions in the treatment of acute Kawasaki syndrome. N Engl J Med.

[15] Newburger JW, Takahashi M, Gerber MA, Gewitz MH, Tani LY, Burns JC, Shulman ST, Bolger AF, Ferrieri P, Baltimore RS, Wilson WR, Baddour LM, Levison ME, Pallasch TJ, Falace DA, Taubert KA (2004). Diagnosis, treatment, and long-term management of Kawasaki disease: a statement for health professionals from the Committee on Rheumatic Fever, Endocarditis, and Kawasaki Disease, Council on Cardiovascular Disease in the Young, American Heart Association. Pediatrics.

[16] Ng YM, Sung RY, So LY, Fong NC, Ho MH, Cheng YW, Lee SH, Mak WC, Wong DM, Yam MC, Kwok KL, Chiu WK (2005). Kawasaki disease in Hong Kong, 1994 to 2000. Hong Kong Med J.

[17] Peng H (2006) The role and molecular mechanism of 25-(OH) D3 and its receptor in the pathogenesis of Kawasaki disease. Wuhan: Huazhong University of Science and Technology, Graduate Department of Tongji Medical College

[18] Scragg R, Sowers M, Bell C (2007). Serum 25-hydroxyvitamin D, ethnicity, and blood pressure in the Third National Health and Nutrition Examination Survey. Am J Hypertens.

[19] Shimizu C, Oharaseki T, Takahashi K, Kottek A, Franco A, Burns JC (2013). The role of TGF-beta and myofibroblasts in the arteritis of Kawasaki disease. Hum Pathol.

[20] Suzuki Y, Ichiyama T, Ohsaki A, Hasegawa S, Shiraishi M, Furukawa S (2009). Anti-inflammatory effect of 1alpha,25-dihydroxyvitamin D(3) in human coronary arterial endothelial cells: implication for the treatment of Kawasaki disease. J Steroid Biochem Mol Biol.

[21] Valtuena J, Breidenassel C, Folle J, Gonzalez-Gross M (2011). Retinol, beta-carotene, alpha-tocopherol and vitamin D status in European adolescents; regional differences an variability: a review. Nutr Hosp.

[22] van de Luijtgaarden KM, Voute MT, Hoeks SE, Bakker EJ, Chonchol M, Stolker RJ, Rouwet EV, Verhagen HJ (2012). Vitamin D deficiency may be an independent risk factor for arterial disease. Eur J Vasc Endovasc Surg.

[23] van Etten E, Mathieu C (2005). Immunoregulation by 1,25-dihydroxyvitamin D3: basic concepts. J Steroid Biochem Mol Biol.

[24] Weng KP, Hsieh KS, Huang SH, Ou SF, Lai TJ, Tang CW, Lin CC, Ho TY, Liou HH, Ger LP (2013). Interleukin-18 and coronary artery lesions in patients with Kawasaki disease. J Chin Med Assoc.

[25] White JH (2012). Vitamin D metabolism and signaling in the immune system. Rev Endocr Metab Disord.

[26] Wu KM (2012). Vitamin D, calcium nutrition supplement China children. J Pract Pediatr.

[27] Yin W, Wang X, Ding Y, Peng H, Liu YL, Wang RG, Yang YL, Xiong JH, Kang SX (2011). Expression of nuclear factor-kappaBp65 in mononuclear cells in Kawasaki disease and its relation to coronary artery lesions. Indian J Pediatr.

[28] Zhang X, Zhang Z, Liu S, Sun J (2012). Epidemiologic survey of Kawasaki disease in Jilin from 1999 through 2008. Pediatr Cardiol.

